# Enhanced Osteogenic Differentiation of Pluripotent Stem Cells via γ-Secretase Inhibition

**DOI:** 10.3390/ijms22105215

**Published:** 2021-05-14

**Authors:** Summer A. Helmi, Leili Rohani, Ahmed R. Zaher, Youssry M. El Hawary, Derrick E. Rancourt

**Affiliations:** 1 Department of Biochemistry and Molecular Biology, University of Calgary, Calgary, AB T2N 1N4, Canada; summer.helmi@ucalgary.ca; 2 Department of Oral Biology, Faculty of Dentistry, Mansoura University, Mansoura 35516, Egypt; ahmedrz2016@hotmail.com (A.R.Z.); Yhawary2007@mans.edu.eg (Y.M.E.H.); 3 Department of Medicine, School of Biomedical Engineering, The University of British Columbia, Vancouver, BC V6T 1Z3, Canada; leili.rohani@ubc.ca

**Keywords:** embryonic stem cells, iPS, notch signaling, osteogenic differentiation, regenerative medicine

## Abstract

Bone healing is a complex, well-organized process. Multiple factors regulate this process, including growth factors, hormones, cytokines, mechanical stimulation, and aging. One of the most important signaling pathways that affect bone healing is the Notch signaling pathway. It has a significant role in controlling the differentiation of bone mesenchymal stem cells and forming new bone. Interventions to enhance the healing of critical-sized bone defects are of great importance, and stem cell transplantations are eminent candidates for treating such defects. Understanding how Notch signaling impacts pluripotent stem cell differentiation can significantly enhance osteogenesis and improve the overall healing process upon transplantation. In Rancourt’s lab, mouse embryonic stem cells (ESC) have been successfully differentiated to the osteogenic cell lineage. This study investigates the role of Notch signaling inhibition in the osteogenic differentiation of mouse embryonic and induced pluripotent stem cells (iPS). Our data showed that Notch inhibition greatly enhanced the differentiation of both mouse embryonic and induced pluripotent stem cells.

## 1. Introduction

Stem cell therapy is an attractive alternative approach used for bone repair. The involvement of stem cells in the healing process is critical for complicated non-union fractures resulting from trauma, blood insufficiency, and defects associated with chronic diseases that significantly impact the healing process, such as diabetes and osteoporosis [1,2]. 

Loading scaffolds with bone marrow-derived mesenchymal stem cells (MSC) enhanced bone defect healing compared to using the scaffold without MSC [3,4,5]. While successful, the MSC harvesting process, the number of cells harvested, and the tendency of this type of cells to senesce after a limited number of passages made the search for alternative regenerative cells necessary [6].

Embryonic stem cells (ESC) can differentiate into all three germ layers. The high proliferation potential and the tremendous self-renewal ability give these cells an advantage over MSC. ESC have been applied for bone regeneration and proved to be an ideal cell source for bone repair [7,8]. However, the derivation of human ESC and the ethical debate around the destruction of human embryos make these cells’ clinical use impossible [9]. Accordingly, induced pluripotent stem cells (iPS) became an alternative to overcome the disadvantages of ESC. Takahashi and Yamanaka used four transcription factors (Oct4, Sox2, Klf4, and c-Myc) to reprogram fibroblasts back to pluripotency. The produced cell line had the same enormous differentiation capacity as ESC [10]. Moreover, iPS bypass the ethical dilemma of destroying human embryos and can be derived from patients to overcome graft rejection. All the criteria mentioned above make iPS a superior cell transplant choice to enrich scaffolds used for bone repair [11,12].

The osteogenic differentiation of bone progenitor cells and bone marrow mesenchymal stem cells (MSC) results from the interaction between different types of cell signaling. For example, endocrine signaling like hormones, such as parathyroid hormone (PTH) [13], sex hormones [14,15], and glucocorticoids [16,17]. Besides, paracrine and autocrine signaling are also involved in the differentiation process through local growth factors like bone morphogenetic proteins (BMP), transforming growth factor-beta (TGF-β), and fibroblast growth factor-2 (FGF-2) [1,18,19]. The type of interaction causes a cascade of intercellular and intracellular events that result in the differentiation of MSC and osteogenic progenitors to become bone [20].

The Notch signaling pathway has fundamental roles in several developmental processes. This signaling pathway acts in a paracrine manner wherein a ligand-bearing cell signals a receptor-bearing neighbor. This way, Notch signaling maintains and regulates stem cells, cell differentiation, and cellular homeostasis. It contributes to the regulation of tissue balance and the maintenance of stem cells in adults. It also regulates cell differentiation, proliferation, survival, and apoptosis. These essential functions take place through “lateral inhibition” and “boundary induction” [21,22]. In “lateral inhibition”, Notch signaling contributes to binary cell fate choices in cells, which are developmentally equivalent. This mechanism occurs by the inhibition of one fate in some cells and allowing them to adopt another different fate, which means that the cell that adopts the alternative fate blocks this choice in its neighbors [23,24]. “In boundary induction”, Notch signaling induces new cell fates rather than selecting from two alternative ones [21,25].

Canonical Notch signaling consists of the Notch receptors Notch1, Notch2, Notch3, and Notch4. These receptors are transmembrane proteins that bind to Notch ligands Jagged1, Jagged2, Delta-like1, Delta-like3, and Delta-like 4 of the neighboring cells. Ligand-receptor binding promotes a proteolytic reaction where a TACE/ADAM10 and γ-secretase presenilins cleave the Notch intracellular domains (NICD) [26]. NICD then translocates to the nucleus to bind to DNA-binding protein CSL (CBF1/Suppressor of Hairless/LAG-1) to regulate the target genes. The most popular of these target genes are the basic helix-loop-helix (bHLH) transcriptional repressors of the Hairy enhancer of split (HES) and Hairy-related (Hrt) protein families (HEY) [27]. While Notch activation promotes stem cell self-renewal [28], Notch function depends on the stage of development, cell type, and cell state [22,29].

Several studies described the role of Notch signaling in osteoblast fate determination of MSC in the literature with opposing results. Some studies concluded that Notch signaling was essential for stem cell renewal and osteoblast progenitor pool maintenance. Osteoblast differentiation increased upon inhibition of some or all of the Notch signaling cascade. Moreover, Notch activation impaired the differentiation, maturation, and matrix production of osteoblasts [30,31,32]. On the other hand, the results of various in vitro and in vivo studies indicated that activation of Notch signaling enhanced the differentiation of MSC and precursor cell lines [33]. Notch activation also improved matrix mineralization and bone remodeling in animal models [34,35,36]. 

These studies proposed that the effects of Notch signaling in bone regeneration are cell context-dependent. The Notch signaling pathway has different outcomes depending on the differentiation status of target cells and the niche's nature wherein the cells self-renew and differentiate in terms of bone health or disease. However, how Notch carries out such diverse roles in bone cells remains elusive. In the current study, we used DAPT γ-secretase inhibitor to inhibit the Notch signaling pathway and observed the consequent effects on the differentiation of embryonic and induced pluripotent stem cells to osteoblasts.

## 2. Results

### 2.1. Notch Inhibition Synergizes Dexamethasone Mediated More Than Vitamin D Mediated Osteogenic Differentiation

**Figure 1:**
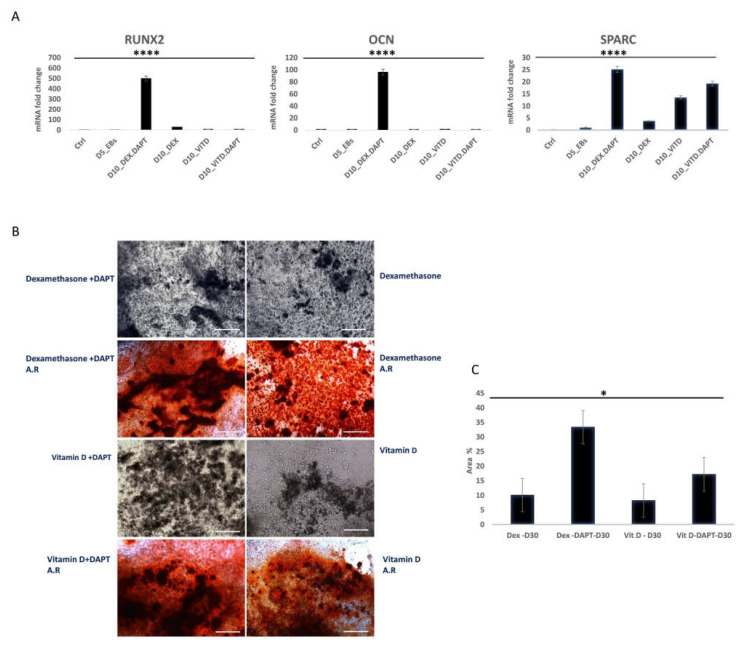
Evaluation of differentiation with Dexamethasone (DEX) and Vitamin D (VITD) based osteogenic media combined with DAPT Notch inhibitor of D3 ES cells. (**A**) Osteogenic gene expression with different media types at 10 days of osteogenic differentiation for the genes RUNX2 (*p* < 0.0001), Osteocalcin (OCN) (*p* < 0.0001), and SPARC (Osteonectin) (*p* < 0.0001) (*n* = 3). Dexamethasone with DAPT (D10-DEX.DAPT), Dexamethasone without DAPT (D10-DEX), vitamin D3 with DAPT (D10-VITD.DAPT), and vitamin D3 without DAPT (D10-VITD). (**B**) Alizarin Red staining (A.R) for cell cultures on different types of osteogenic media at day 30 of osteogenic differentiation, scale bar 100 µm. (**C**) Quantification of surface area positive for Alizarin Red staining (*p* < 0.05). * = *p* < 0.05, **** = *p* < 0.0001.

We compared two osteogenic differentiation media combined with DAPT Notch inhibitor to optimize the differentiation protocol used in this study. DAPT efficiently blocks the presenilin–γ-secretase complex [37] and prevents Notch signaling activation [38,39]. On day 10 of osteogenic differentiation, both differentiation media showed a significant increase in bone marker gene expression compared to controls and cell cultures where DAPT was absent. D3 embryoid bodies (EBs) cultured in Dexamethasone (DEX) combined with DAPT showed higher expression of the early osteogenic marker RUNX2 (*p* < 0.0001) and the late osteogenic markers OCN (*p* < 0.0001) and SPARC (Osteonectin) (*p* < 0.0001) compared to cultures that received both vitamin D (VITD) and vitamin D combined with DAPT (Figure 1A). To confirm these results, on day 30 of osteogenic differentiation, cells cultured in both types of osteogenic media combined with DAPT were fixed and stained with Alizarin Red stain to confirm the differentiation of D3 cells to active osteoblasts capable of the formation of calcified bone matrix. The calcified matrix was stained in red. We captured images of the stained cultures, and the stained surface area was calculated using ImageJ software. Cells differentiated under the effect of Dexamethasone and DAPT showed wider stained surface area than cells differentiated under the impact of vitamin D combined with DAPT and control cultures that did not receive the Notch inhibitor (*p* = 0.03) (Figure 1B,C). Based on the previous results, we continued to use dexamethasone-based media for the rest of this study.

### 2.2. Effect of Notch Inhibition on the Bone Marker Gene Expression during the Osteogenic Differentiation of Embryonic and Induced Pluripotent Stem Cells

**Figure 2:**
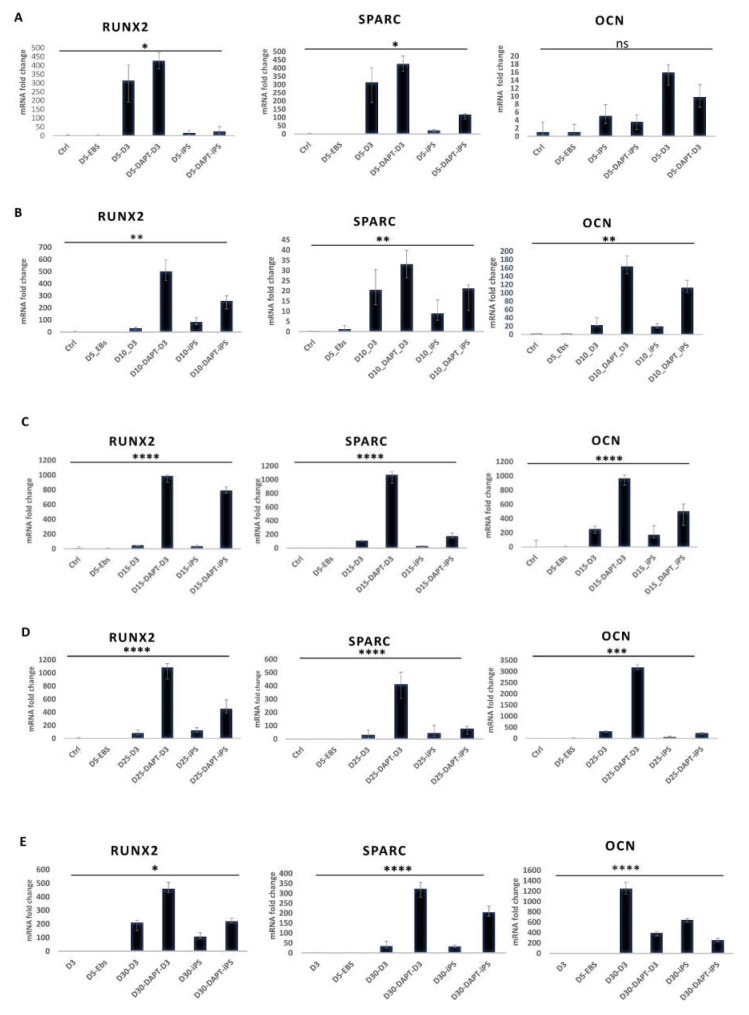
Gene expression on days 5 to 30 of osteogenesis. (**A**) Gene expression on day 5 of osteogenesis, Dexamethasone in combination with DAPT in D3 ESC cells (D5-DAPT-D3) and iPS (D5-DAPT-iPS), Dexamethasone alone (D5-D3), (D5-iPS) for RUNX2 (*p* < 0.05), SPARC (*p* < 0.05), OCN (*p* > 0.05). (**B**) Gene expression on day 10 of osteogenesis. Dexamethasone in combination with DAPT in D3 cells (D10-DAPT-D3) and iPS (D10-DAPT_iPS), Dexamethasone alone (D10-D3), (D10-iPS) for RUNX2 (*p* < 0.01), SPARC (*p* < 0.01), OCN (*p* < 0.01). (**C**) Gene expression on day 15, Dexamethasone in combination with DAPT in D3 (D15-DAPT-D3) and iPS (D15-DAPT-iPS), Dexamethasone alone (D15-D3), (D15-iPS) for RUNX2-(*p* < 0.0001), SPARC (*p* < 0.0001), OCN (*p* < 0.0001). (**D**) Gene expression on day 25: Dexamethasone in combination with DAPT D3 (D25-DAPT-D3) and iPS (D25-DAPT-iPS), Dexamethasone alone (D25-D3), (D25-iPS) for RUNX2-(*p* < 0.0001), SPARC (*p* < 0.0001), OCN (*p* < 0.001). (**E**) Gene expression on day 30 of osteogenesis. Dexamethasone in combination with DAPT in D3 (D30-DAPT-D3) and iPS (D30-DAPT-iPS), Dexamethasone alone (D30-D3), (D30-iPS) for RUNX2-(*p* < 0.05), SPARC (*p* < 0.0001), OCN (*p* < 0.0001) (*n* = 3). * = *p* < 0.05, ** = *p* < 0.01, *** = *p* < 0.001, **** = *p* < 0.0001.

To demonstrate the effect of Notch inhibition on the various time points during osteogenic differentiation of embryonic and induced pluripotent stem cells, we performed qRT-PCR starting on day 5 and up to day 30 with five-day intervals in between. Gene expression fold changes of the early marker RUNX2 and the late markers OCN and SPARC were quantified. On day 5 of differentiation, Notch inhibition increased the expression of RUNX2 (*p* = 0.02) and SPARC (*p* = 0.03) for embryonic and induced pluripotent stem cells, while this effect was not demonstrated for the late marker OCN (*p* = 0.1). On day 10, the difference in expression of RUNX2 and SPARC increased in the Notch inhibition groups in both D3 ESC and iPS (*p* = 0.007), compared to the groups with no inhibition. At this time point, OCN gene expression was significantly increased in both cell lines with Notch inhibition compared to the groups where no inhibitor was added (*p* = 0.002) (Figure 2A,B).

As the differentiation process progressed, on days 15 and 25, gene expression fold change continued to be higher in the D3 and iPS samples where the Notch inhibitor was added for the genes RUNX2 (*p* < 0.0001 on day 15, *p* < 0.0001 on day 25), SPARC (*p* < 0.0001 on day 15, *p* < 0.0001on day 25), and OCN (*p* < 0.0001 on 15 days, *p* = 0.0003 on day 25 ) when compared to the samples without the Notch inhibitor (Figure 2C,D). Moving forward, and on day 30 of osteogenesis and as the differentiation process reached a late stage, the D3 and iPS samples where the Notch inhibitor was added showed higher expression of RUNX2 (*p* = 0.01) and SPARC (*p* < 0.0001) when compared to the samples where no Notch inhibition took place. This was not the case for OCN; Notch inhibition did not increase OCN expression at this point for the cell lines under investigation (*p* < 0.0001) (Figure 2E).

### 2.3. Detection of the Effect of Notch Inhibition on Bone Protein Expression during the Osteogenic Differentiation Process

**Figure 3:**
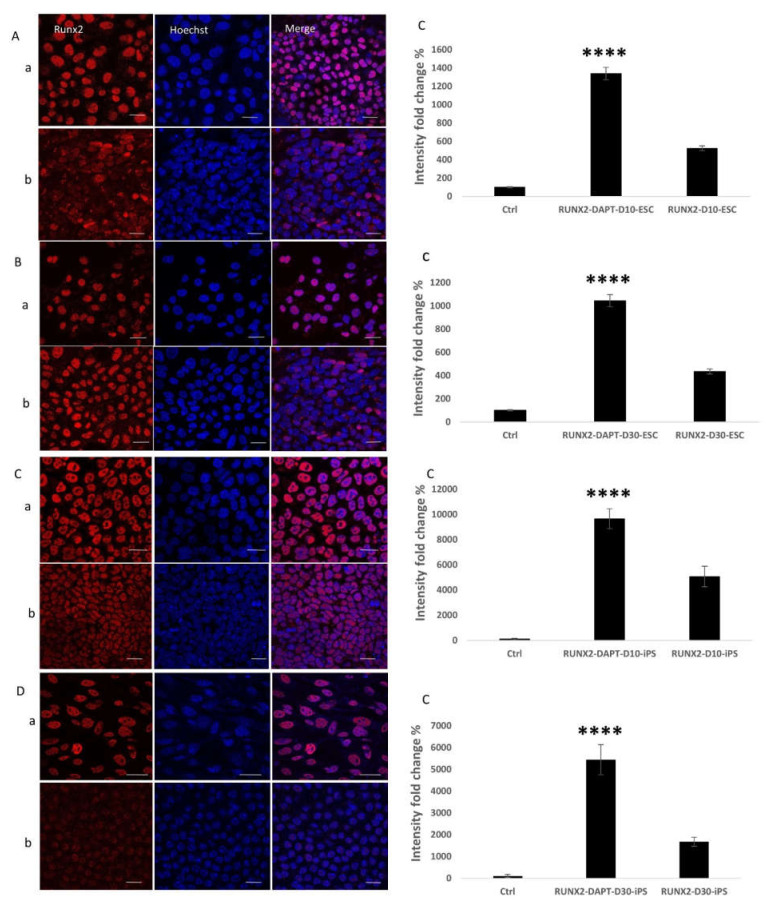
Immunofluorescent staining against the early bone marker RUNX2. (**A**) RUNX2 expression on day 10 of osteogenic differentiation in ESC. (**B**) RUNX2 expression on day 30 of osteogenic differentiation in ESC. (**C**) RUNX2 expression on day 10 of osteogenesis in iPS. (**D**) RUNX2 expression on day 30 of osteogenesis in iPS. In all the previous figures: (**a**) Cell cultures where DAPT Notch inhibitor is added, (**b**) cell cultures where DAPT Notch inhibitor is absent, (**c**) Quantification of fluorescence intensity and comparison between the two cell culture conditions (with and without DAPT); (**A**-**c**) (*p* < 0.0001), (**B**-**c)** (*p* < 0.0001), (**C**-**c)** (*p* < 0.0001), (**D**-**c**) (*p* < 0.0001); Scale bar 20 µm. **** = *p* < 0.0001.

**Figure 4:**
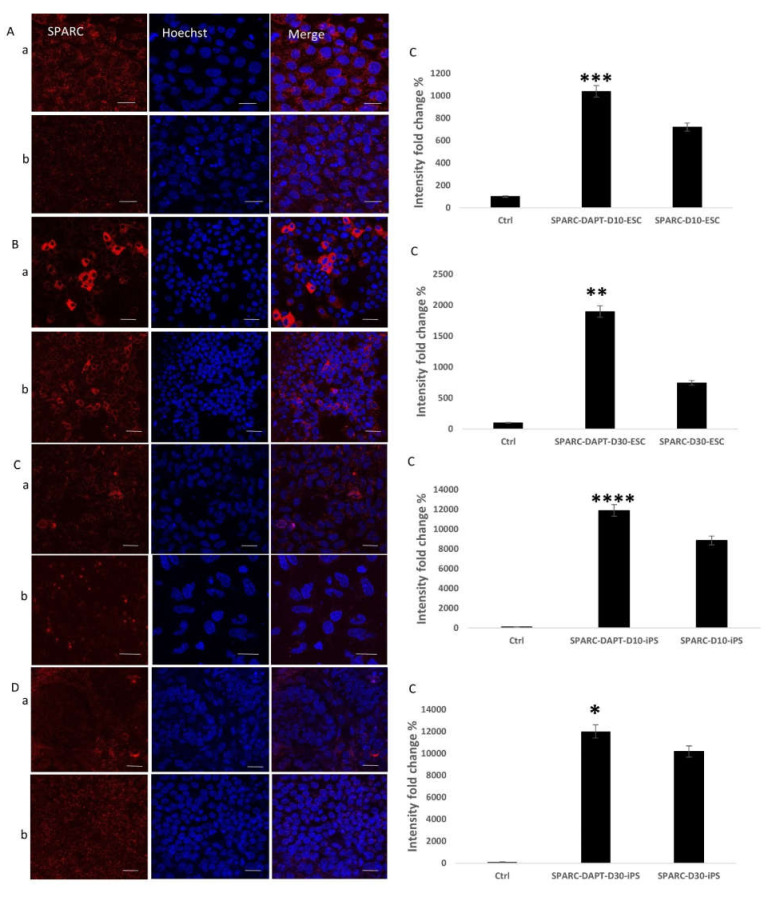
Immunofluorescent staining against the late bone marker SPARC. (**A**) SPARC expression on day 10 of osteogenic differentiation in ESC. (**B**) SPARC expression on day 30 of osteogenic differentiation in ESC. (**C**) SPARC expression on day 10 of osteogenesis in iPS. (**D**) SPARC expression on day 30 of osteogenesis in iPS. In all the previous figures: (**a**) Cell cultures where DAPT Notch inhibitor is added, (**b**) cell cultures where DAPT Notch inhibitor is absent. (**c**) Quantification of fluorescence intensity and comparison between the two cell culture conditions (with and without DAPT); (**A**-**c**) (*p* < 0.001), (**B**-**c**) (*p* < 0.01), (**C**-**c**) (*p* < 0.0001), (**D**-**c**) (*p* < 0.05); Scale bar 20 µm. * = *p* < 0.05, ** = *p* < 0.01, *** = *p* < 0.001, **** = *p* < 0.0001

**Figure 5:**
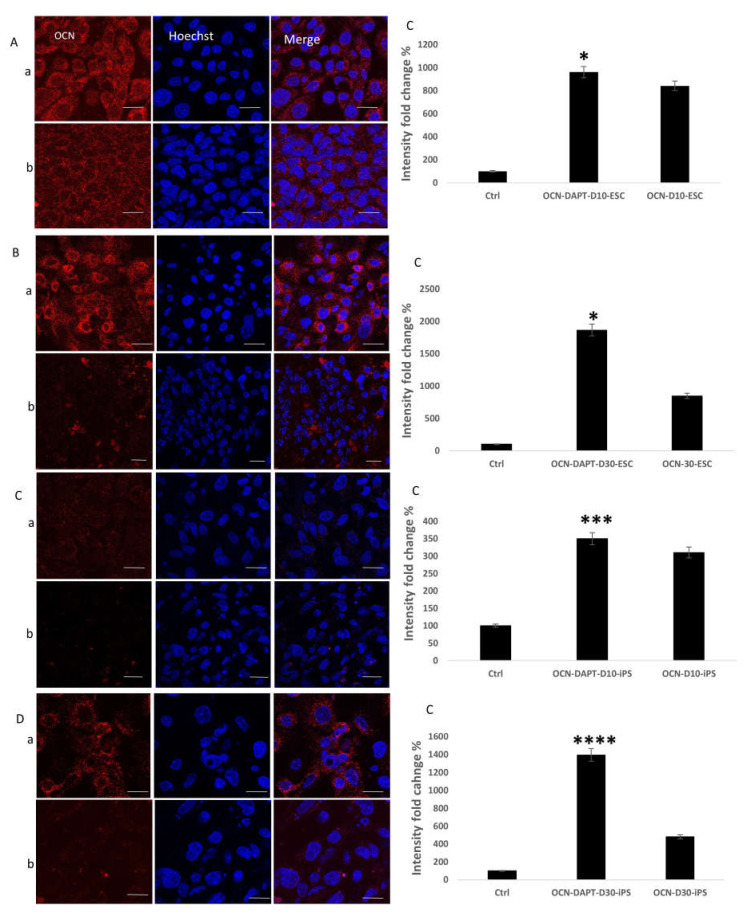
Immunofluorescent staining against the late bone marker OCN. (**A**) OCN expression on day 10 of osteogenic differentiation in ESC and IPS. (**B**) OCN expression on day 30 of osteogenic differentiation in ESC. (**C**) OCN expression on day 10 of osteogenesis in IPS. (**D**) OCN expression on day 30 of osteogenesis in iPS. In all the previous figures: (**a**) Cell cultures where DAPT Notch inhibitor is added. (**b**) cell cultures where DAPT Notch inhibitor is absent. (**c**) Quantification of fluorescence intensity and comparison between the two cell culture conditions (with Notch inhibitor and the absence of Notch inhibitor), (**A-c**) (*p* < 0.05), (**B-c**) (p < 0.05), (**C-c**) (*p* < 0.001), (**D-c**) (*p* < 0.0001); Scale bar 20 µm. * = *p* < 0.05, *** = *p* < 0.001, **** = *p* < 0.0001.

To identify the effect of Notch inhibition on bone protein expression and to further confirm the previous results, immunofluorescent staining and confocal microscopy were applied to the early differentiated ESC and iPS cell cultures on day 10 and the late differentiated cell cultures on day 30. The staining was against the early osteogenic marker RUNX2 (Figure 3) and the late markers SPARC (Osteonectin) (Figure 4), and Osteocalcin (OCN) (Figure 5). For early cultures, fluorescence quantification showed increased expression of the RUNX2 (*p* < 0.0001 for ESC, *p* < 0.0001 for iPS), SPARC (*p* = 0.0004 for ESC, *p* < 0.0001 for iPS), and OCN (*p* = 0.02 for ESC, *p* = 0.0002 for iPS) when compared to cultures where Notch inhibition was absent for ESC and iPS. On day 30, fluorescence quantification revealed the same augmented effect regarding the expression of the three proteins RUNX2 (*p* < 0.0001 for ESC, *p* < 0.0001 for iPS), OCN (*p* = 0.01 for ESC, *p* < 0.0001 for iPS), and SPARC (*p* = 0.002 for ESC, *p* = 0.03 for iPS) in the cell cultures that received the Notch inhibitor. This outcome was observed in both ESCs and iPS.

### 2.4. Detection of Mesodermal Differentiation of ESC and iPS

**Figure 6:**
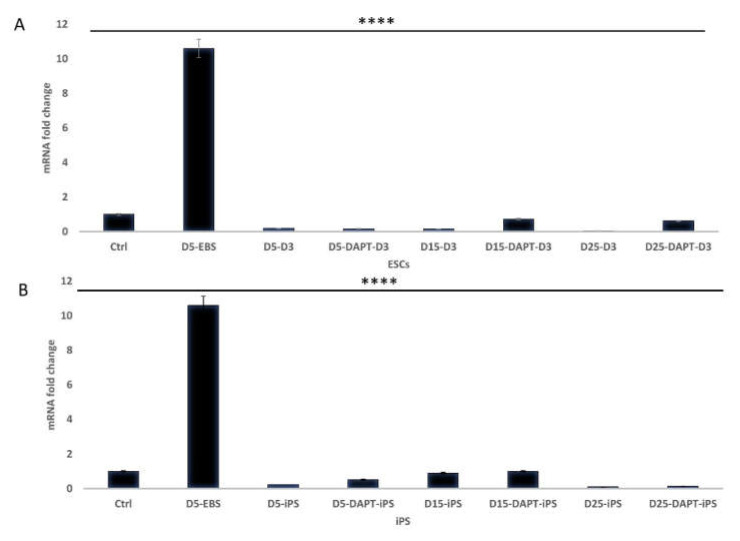
Detection of the mesodermal marker Brachyury. (**A**) expression of Brachyury in differentiating ESC; Embryoid bodies at 5 days (D5-Ebs), 5 days of osteogenic differentiation without DAPT (D5-D3), 5 days with DAPT (D5-DAPT-D3), 15 days without DAPT (D-15-D3), 15 days with DAPT (D15-DAPT-D3), 25 days without DAPT (D25-D3), 25 days with DAPT (D25-DAPT-D3) (*p* < 0.0001). (**B**) expression of Brachyury in differentiating iPS; Embryoid bodies at 5 days (D5-Ebs), 5 days of osteogenic differentiation without DAPT (D5-iPS), 5 days with DAPT (D5-DAPT-iPS), 15 days without DAPT (D-15-iPS), 15 days with DAPT (D15-DAPT-iPS), 25 days without DAPT (D25-iPS), 25 days with DAPT (D25-DAPT-iPS) (*p* < 0.0001) (*n* = 3). **** = *p* < 0.0001.

As bone is mesodermal in origin, we detected mesoderm formation during the osteogenic differentiation process (Figure 6). qRT-PCR showed the highest expression of the early mesodermal marker Brachyury during the embryoid body stage. Expression levels decreased as differentiation progressed to the osteogenic fate. We found that in both ESC (*p* < 0.0001) and iPS (*p* < 0.0001), Notch inhibition heightened the expression of Brachyury on days 5, 15, and 25.

### 2.5. Expression of HES1 and HEY1 as Notch Target Genes during Osteogenesis

**Figure 7:**
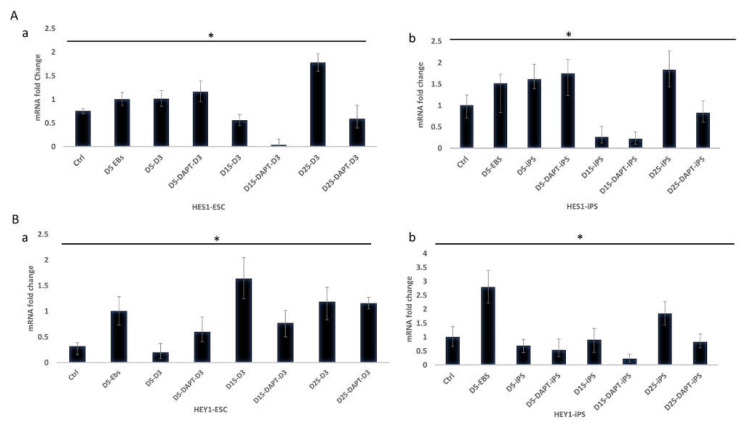
Effect of DAPT on the expression of Notch target genes HES1 and HEY1 in ESC and iPS cells on days 5, 15, and 25 of differentiation. (**A**) (**a**) HES1 Expression in D3 cells, (**b**) HES1 Expression in iPS. On day 5 (D5-DAPT-D3), (D5-DAPT-iPS), on day 15 (D15-DAPT-D3), (D15-DAPT-iPS), and day 25 (D25-DAPT-D3), (D25-DAPT-iPS) (*p* < 0.05) (*n* = 3). (**B**); (**a**) HEY1 expression in D3 cells. (**b**) HEY1 expression in iPS at day 5 (D5-DAPT-D3), (D5-DAPT-iPS), at ay 15 (D15-DAPT-D3), (D15-DAPT-iPS), and day 25 (D25-DAPT-D3), (D25-DAPT-iPS) (*p* < 0.05) (*n* = 3). * = *p* < 0.05.

qRT-PCR detected the expression changes of HES1 and HEY1 during the various differentiation stages (Figure 7). HES1 (Figure 7A) and HEY1(Figure 7B) were expressed in undifferentiated cells and EBs of ESC and iPS. On day 5, the expression of HES1 and HEY1 did not seem to be affected by γ-secretase inhibition, and the expression levels were nearly similar in both ESC and iPS. At later time points on days 15 and 25, expression levels were significantly decreased by Notch inhibition. This observation was attained in ESC (*p*=0.02) and iPS (*p* = 0.02).

## 3. Discussion

Improving the differentiation process of pluripotent stem cells through understanding the signaling cues that control and contribute to the differentiation process to the desired cell type, in addition to the correct choice of the transplantation scaffold, can yield a successful nearby clinical therapy for a wide variety of tissue and organ defects [40,41,42].

The Notch signaling pathway plays a substantial role in the differentiation of iPS to several adult cell types. Notch inhibition during the early stage of iPS differentiation to blood cells caused a significant decrease in mature erythrocytes in cultures [43]. Notch activation during the differentiation of induced pluripotent stem cells derived from patients with hypoplastic left heart syndrome (HLHS) restored their cardiomyocyte differentiation capacity and beating rate. It suppressed smooth muscle cell formation [44]. On the other hand, Notch inhibition improved the differentiation of iPS to neural stem cells [45]. Moreover, Notch inhibition accelerated the neuronal generation of pluripotent stem cells in cell cultures and after transplantation to treat spinal cord injury [46]. Moreover, timely inhibition of Notch signaling in synergy with ascorbic acid promoted cardiomyocytes' differentiation from induced pluripotent stem cells [47].

Notch signaling proved to play an essential role in skeletal development and bone remodeling [48]; furthermore, this signaling pathway is critical for skeletal stem cell differentiation and renewal [16]. In the current study, we investigated the role of Notch inhibition on the osteogenic differentiation of embryonic and induced pluripotent stem cells. Our data revealed that the knockdown of the Notch signaling pathway via γ-secretase inhibition enhanced mouse embryonic and induced pluripotent stem cell differentiation and commitment to the osteogenic fate. 

Consistent with our results, several reports have indicated that the inhibition of Notch signaling regulated in vitro osteogenic differentiation from various progenitor and immature cell types. A study conducted in vitro revealed that Notch1 decreased osteoblast precursor cell differentiation [49]. Notch1 activation in mesenchymal stem cells and mature osteoblasts caused severe osteopenia and resulted in defective bone structure formation. Besides, the deletion of Notch1 and Notch2 in osteoblast progenitor cells resulted in increased osteoblast number and cancellous bone formation [31]. Notch1 inhibition also decreased the proliferation yet promoted the osteogenic differentiation of bone marrow mesenchymal stem cells [50]. Other studies reported how γ-secretase inhibition restored the osteogenic differentiation capacity of aged bone marrow stem cells in mice [51] which is similar to the reinstated effect γ-secretase inhibition had on human skeletal mesenchymal stem cells used for ectopic bone formation in mice [52].

Dexamethasone and 1α,25-dihydroxy vitamin D3 (VITD) are the principal and standard components of osteogenic cell culture media [53]. Our results showed that combining DAPT Notch inhibitor with Dexamethasone resulted in a synergistic effect on ESC and iPS osteogenic differentiation compared with combining VITD and DAPT (Figure 1). Dexamethasone was demonstrated to stimulate the differentiation of stem cells to osteoblasts through multiple mechanisms. These mechanisms include the activation of RUNX2 expression through wnt/β-catenin pathway activation. Dexamethasone also increases RUNX2 phosphorylation by the mitogen-activated protein kinase (MAPK).

Moreover, Dexamethasone activates RUNX2 transcription through TAZ activation (transcriptional coactivator with PDZ-binding motif) [54]. On the other hand, some studies have demonstrated that VITD suppresses RUNX2 expression in mouse cells by binding to vitamin D receptors in the nucleus [55]. While both components proved to decrease Notch receptor expression in differentiating cells [56], which in turn enhances RUNX2 expression through the inactivation of the Notch target gene HEY-1, Dexamethasone appears to have the most potent effect on the expression of RUNX2, which in turn, activates the expression of many other osteogenic genes. These findings seem to explain its powerful impact on the osteogenic differentiation process when combined with DAPT.

qPCR results showed significantly increased expression of the osteogenic gene RUNX2 in ESC and iPS at various time points in the cell cultures that received DAPT compared to the cultures where DAPT was absent. These results came in agreement with the fact that the Notch1 and Notch2 receptor inhibition suppressed Notch target genes HES1, HEY1 which are major inhibitors of RUNX2 activation [31,57]. Similarly, OCN expression levels were enhanced by Notch inhibition as differentiation progressed. We suggest that this improvement is subsequent to RUNX2 improved expression [58]. Another mechanism contributing to OCN expression's advancement might be glycolysis stimulation by inhibiting the Notch signaling pathway since OCN expression is highly dependent on glycolysis [32,59,60].

SPARC, also known as Osteonectin, is a protein produced by mature osteoblasts and some unmineralized tissue cells [61]. In bone, this protein is associated with the production of type I collagen; SPARC contains a collagen-binding domain and a hydroxyapatite binding region, which allows this protein to bind collagen and hydroxyapatite crystals and release calcium ions, which is essential for the mineralization of the collagen matrix in bones [62]. Our results demonstrated that Notch inhibition maintained a stimulatory effect on the gene expression of SPARC during the course of the experiment. To our knowledge, the mechanism wherein Notch signaling enhances SPARC expression during bone formation is unknown. However, our results are aligned with other studies suggesting that Notch inhibition increased SPARC expression in neuroblastoma, astrocytoma, and medulloblastoma. Our studies indicate that the stimulatory effect that Notch inhibition had on SPARC gene expression results from suppressing HES1 and HEY1 [63,64,65]. Further investigation is required to determine if enhancing SPARC expression by inhibiting Notch during the osteogenic differentiation occurs via the exact mechanism.

As bone is mesodermal in origin, we tested the mesodermal marker Brachyury expression [66] and investigated how Notch inhibition affected its expression. The results demonstrated a higher expression level in pluripotent stem cell-derived embryoid bodies. During stochastic differentiation to all three germ layers, Brachyury expression levels decreased as differentiation progressed [67]. Our observation is corroborated by a previous study indicating that Notch's activation led to disruption in some mesodermal precursors’ differentiation [68]. Likewise, Other studies suggested that Notch 1 inhibition upregulated Brachyury expression and improved cardiac differentiation of embryonic stem cells [69,70]. This improvement in expression can be another contributing factor to the overall augmenting effect that Notch inhibition had on osteogenic differentiation. Immunofluorescent imaging revealed results that strengthened the qPCR results. Fluorescence quantification showed increased expression of the early marker RUNX2 and the late markers SPARC and OCN at early differentiation (10 days) and late differentiation (30 days). 

For further confirmation of the above results, we tested the expression of HES1 and HEY1 with DAPT application. We observed a decreased expression of HES1 and HEY1 in ESC and iPS on days 15 and 25 in response to DAPT application. Surprisingly, we noticed an increased expression of both genes on day 5 of application. Other studies reported that the expression levels of the Notch target Genes HES1, HEY1, HEY2 increased despite DAPT application at the early stages of inhibition [71,72]. They suggested that the BMP-SMAD1/5 pathway had synergistic action on the Notch signaling pathway independent of γ-secretase at the early differentiation stage [73]. 

To our knowledge, there are very few studies that investigate how Notch signaling affects the differentiation of embryonic and induced pluripotent stem cells to the osteogenic lineage. Our results demonstrated the augmenting effect that Notch inhibition had on the osteogenic differentiation of embryonic and induced pluripotent stem cells on the transcription and translation levels. Here, we shed light on the synergistic effect that the combination of Dexamethasone and DAPT had on the differentiation process. Moreover, our results demonstrated that enhanced mesodermal differentiation might be another element to consider contributing to improving the differentiation process's outcome. We suggest that additional studies are needed to fully understand the crosstalk among the different signaling pathways that control stem cell differentiation to bone cells. Further studies need to be accomplished to promote the differentiation outcome of embryonic and induced pluripotent stem cells to bring stem cell-based therapies to fruition.

## 4. Materials and Methods

### 4.1. Cell Lines and Cultures

#### 4.1.1. Pluripotent Stem Cells

Mouse embryonic (D3 line) [74] and induced pluripotent (miPS line) [75] stem cells were used for this study. D3 and miPS cells were maintained in the pluripotent state in high glucose DMEM (Gibco, Burlington, ON, Canada) supplemented with 15% FBS, 1% non-essential amino acids (Invitrogen, Burlington, ON, Canada), 50 U/ mL Penicillin, and 50 µg/mL Streptomycin (Invitrogen, Burlington, ON, Canada), 0.1 mM β-mercaptoethanol (Gibco) and 1000 U/mL Leukemia Inhibitory Factor (LIF). Pluripotent cultures were sub-cultured every second day on murine embryonic fibroblast feeder cells (MEFs).

#### 4.1.2. Embryoid Body (EB) Formation

D3 and miPS embryoid bodies were formed using the hanging drop method. [76] Day 2 EBs were transferred to differentiation media composed of DMEM (Gibco, Burlington, ON, Canada), supplemented with 15% FBS (Gibco), 1% non-essential amino acids (Invitrogen, Burlington, ON, Canada), 50 U/mL Penicillin and 50 µg/mL Streptomycin (Invitrogen, Burlington, ON, Canada), 0.1 mM β-mercaptoethanol (Gibco, Burlington, ON, Canada) for three days before transitioning to osteogenic differentiation [77].

#### 4.1.3. Osteogenic Differentiation

For vitamin D3 and dexamethasone-based differentiation protocols, day 5 EBs were transferred to gelatin-coated cell culture dishes containing osteoblast differentiation media. The media composed of β-glycerophosphate (10 mM) (Sigma, High River, AB, Canada), ascorbic acid (50 µg/mL) (Sigma, High River, AB, Canada), and either 1,25-OH2 vitamin D3 or Dexamethasone (5 × 10^−8^M) (Sigma, High River, AB, Canada) starting at day 5 and up to day 30.

### 4.2. Notch Pathway Inhibition

Notch γ-secretase inhibitor DAPT (*N*-[*N*-(3,5-difluorophenacetyl)-l-alanyl]-*S*-phenyl glycine t-butyl ester) (10 µM) (Roche, High River, AB, Canada) was added to the osteogenic media for both D3 and miPS cells from day 5 to 30 of differentiation [78]. 

### 4.3. Evaluation of Osteogenic Differentiation

#### 4.3.1. Alizarin Red Staining

Day 30 cell cultures were fixed overnight at 4 °C in 4% paraformaldehyde (PFA). The next day, cell cultures were washed 3 times in PBS and placed in 1% KOH solution for 48 hours at 4 °C and subsequently stained with Alizarin Red (1 mg Alizarin Red, 100 mL of 1% KOH) for 48 h at 4 °C. The cultures were rinsed with 1% KOH several times. Afterward, PBS was added for visualization. The stained surface area was measured and analyzed using ImageJ software (National Institutes of Health and the Laboratory for Optical and Computational Instrumentation (LOCI, University of Wisconsin), Bethesda, MD, USA).

#### 4.3.2. qPCR

##### mRNA Isolation and cDNA Synthesis

Cell cultures containing differentiated EBs were isolated at multiple time points (days 5, 10, 15, 25, and 30 of osteogenesis). Cells were isolated using TrypLE Express dissociation reagent (Gibco, Burlington, ON, Canada). mRNA Isolation was performed using PureLink™ RNA Mini Kit (Thermo Fisher Scientific, Calgary, AB, Canada) according to the manufacturer's instructions. mRNA was measured using a Nano Photometer P-Class (IMPLEN, Munich Germany). cDNA synthesis was performed using SuperScript™ IV Reverse Transcriptase (Thermo Fisher Scientific, Calgary, AB, Canada) according to the manufacturer’s instructions.

##### RT-qPCR Analysis 

RT-qPCR was employed to quantify the gene expression levels using TaqMan Gene Expression Assays. For osteogenesis, we used the early maker Runx2 (Assay ID Mm00501584-m1) in addition to late osteogenic markers Osteocalcin (OCN) (Assay ID Mm 03413826-mH) and SPARC (Osteonectin) (Assay ID Mm00486332-m1). For Notch target gene expression, we used HES1 (Probe ID Mm01342805-m1) and HEY1 (Assay ID Mm00468865-m1). For early mesodermal differentiation, we used Brachyury (Assay ID Mm00496699-m1). All TaqMan Gene Expression Assays were obtained from Thermo Fisher Scientific. TaqMan Universal PCR MasterMix No AmpErase (Applied Biosystems) was used according to the manufacturer's instructions. StepOnePlus™ Real-Time PCR System was used for running all the samples with the following program: UNG incubation at 50 °C for 2 min; enzyme activation at 95 °C for 20 s; denaturation at 95 °C for 3 s; annealing was performed for 40 cycles at 60 °C for 30 s (40 cycles). The resulting threshold (Ct) values were analyzed with the ΔΔCt method. GAPDH was used as the reference gene, and undifferentiated D3/miPS cells were used as reference samples. 3 biological replicas and 3 technical replicas of each sample were used for the analysis of this test.

### 4.4. Immunofluorescence

#### 4.4.1. Immunofluorescent Staining

EBs were generated according to the previously described method. On day 5, EBs were seeded on gelatin-coated glass-bottom cell culture dishes Fluorodish (World Precision Instruments, Sarasota, FL, USA). On days 10 and 30 of osteogenic differentiation, cells were washed in PBS and fixed 4% PFA in PBS for 45 min. Cells were washed in PBS and permeabilized using 0.25% Triton X-100 (Sigma, High River, AB, Canada) in PBS for 45 min. Blocking was done using 5% filtered BSA (Thermo Fisher Scientific, Calgary, AB, Canada) in PBS for 4 hours. Primary antibodies to the osteogenic markers Runx2, OCN, and SPARC (Santa Cruz Biotechnology, Dallas, TX, USA) were added, and dishes were kept at 4 °C overnight. The next day, cells were washed in PBS, and the secondary antibody Alexa 568 (Millipore, Etobicoke, ON, Canada) was added to the cells for 10 min at room temperature. After washing the cells in PBS, Hoechst (Millipore, Etobicoke, ON, Canada), in PBS, was added for 10 min at room temperature to stain the nuclei. Cells were washed in PBS then enough fresh PBS was added to keep the cells from drying.

#### 4.4.2. Fluorescence Intensity and Distribution

**Figure 8:**
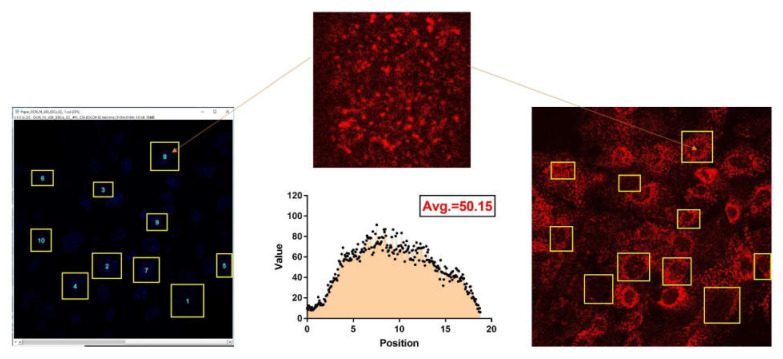
An example of the selection of fluorescent area stained by secondary antibody and representation of fluorescence quantification and distribution.

Ten areas were selected at random in the pictures chosen for analysis, and both intensity and distribution of positive staining were analyzed by ImageJ software. The data were normalized to control measurements (Figure 8).

## 5. Statistical Analysis

For qPCR and quantification of surface area stained with Alizarin Red, one-way ANOVA test was used to compare sample groups. *p* values < 0.05 were considered significant.

For fluorescent intensity analysis, Two-sided paired student's *t*-test was used to compare sample groups. *p* values < 0.05 were considered significant.

* = *p* < 0.05.

** = *p* < 0.01.

*** = *p* < 0.001.

**** = *p* < 0.0001.

ns = non-significant (*p* > 0.05)

## Data Availability

Not applicable.
